# Homeostatic Changes in GABA and Glutamate Receptors on Excitatory Cortical Neurons during Sleep Deprivation and Recovery

**DOI:** 10.3389/fnsys.2017.00017

**Published:** 2017-03-31

**Authors:** Esther del Cid-Pellitero, Anton Plavski, Lynda Mainville, Barbara E. Jones

**Affiliations:** Department of Neurology and Neurosurgery, McGill University, Montreal Neurological InstituteMontreal, QC, Canada

**Keywords:** AMPA, Arc, CaMKIIα, GABA_A_, gamma activity, GluA1

## Abstract

Neuronal activity is regulated in a homeostatic manner through changes in inhibitory GABA and excitatory glutamate (Glu) AMPA (A) receptors (GluARs). Using immunofluorescent staining, we examined whether calcium/calmodulin-dependent protein kinase IIα (CaMKIIα)-labeled (+) excitatory neurons in the barrel cortex undergo such homeostatic regulation following enforced waking with associated cortical activation during the day when mice normally sleep the majority of the time. Sleep deprived mice were prevented from falling asleep by unilateral whisker stimulation and sleep recovery (SR) mice allowed to sleep freely following deprivation. In parallel with changes in c-Fos reflecting changes in activity, (β2-3 subunits of) GABA_A_ Rs were increased on the membrane of CaMKIIα+ neurons with enforced waking and returned to baseline levels with SR in barrel cortex on sides both contra- and ipsilateral to the whisker stimulation. The GABA_A_R increase was correlated with increased gamma electroencephalographic (EEG) activity across conditions. On the other hand, (GluA1 subunits of) AMPA Rs were progressively removed from the membrane of CaMKIIα+ neurons by (Rab5+) early endosomes during enforced waking and returned to the membrane by (Rab11+) recycling endosomes during SR. The internalization of the GluA1Rs paralleled the expression of Arc, which mediates homeostatic regulation of AMPA receptors through an endocytic pathway. The reciprocal changes in GluA1Rs relative to GABA_A_Rs suggest homeostatic down-scaling during enforced waking and sensory stimulation and restorative up-scaling during recovery sleep. Such homeostatic changes with sleep-wake states and their associated cortical activities could stabilize excitability and activity in excitatory cortical neurons.

## Introduction

The activity of individual neurons is known to be regulated in a homeostatic manner, such that following prolonged activity, their excitability is reduced, and reciprocally following prolonged silence, their excitability is increased (Turrigiano, 1999). The sleep-wake cycle, a basic rest-activity cycle (Kleitman, 1939), likely serves in this homeostatic process. Indeed, most neurons through the brainstem and forebrain discharge at their highest rates during waking and lowest or null rates during slow wave sleep (SWS; Jones, 2011). Neurons in the cortex discharge in a different pattern, going from tonic during waking to phasic during SWS with periods of bursting during depolarized (Up) states interposed by periods of silence during hyperpolarized (Down) states, as elucidated through *in vivo* and *in vitro* studies (Steriade et al., 1993, 2001; Sanchez-Vives and McCormick, 2000). This change in firing pattern is reflected in electroencephalographic (EEG) activity by maximal high frequency gamma activity (30–60 Hz) during waking and maximal slow delta activity (0.5–4.0 Hz) during SWS (Maloney et al., 1997). Relative to waking, metabolism is reduced by up to 40% during SWS in the cortex, indicating a state of rest for cortical neurons (Maquet et al., 1990; Vyazovskiy et al., 2008b). The alteration in firing pattern during SWS reflects a homeostatic process since the periods of silence (Off) between periods of fast activity (On) are longest immediately following prolonged waking (Vyazovskiy et al., 2009). Moreover, sleep deprivation (SD) with enforced waking leads to an increase in sleep and cortical slow wave activity with sleep recovery (SR; Borbély et al., 1984; Tobler and Borbély, 1990). This homeostatic process is also evident locally in cortical regions as a function of their prior activity or use, whereby unilateral somatosensory stimulation leads to subsequent increases in slow wave activity in the contralateral somatosensory cortex (Kattler et al., 1994; Vyazovskiy et al., 2000).

In considering the homeostatic nature of sleep, Tononi and Cirelli (2003) formally proposed that sleep serves in synaptic homeostasis, such that net synaptic potentiation occurs with learning during waking and net synaptic depression occurs with slow wave activity during sleep (Vyazovskiy et al., 2008a). However, this hypothesis is incompatible with evidence that synaptic potentiation and consolidation of memories are actually enhanced with slow wave activity and SWS (Aton et al., 2009; Chauvette et al., 2012; Rasch and Born, 2013). Moreover, long term potentiation (LTP) and long term depression (LTD) both occur with learning during waking (Kemp and Manahan-Vaughan, 2007) and such Hebbian plasticity is varied and synapse-specific (Lisman, 1989; Lisman and Harris, 1993; Turrigiano et al., 1998; Ibata et al., 2008). In contrast, as elucidated by Turrigiano (1999), Turrigiano and Nelson (2004), homeostatic synaptic scaling is a global phenomenon which adjusts the scale of all synapses on a neuron depending upon its activity and thus allows the relative weights of different synapses modified by Hebbian plasticity to be maintained. Sleep would more likely involve such global homeostatic scaling or changes in excitability which can occur through global changes in inhibitory, GABA (GABA_A_) receptors and excitatory, glutamate (Glu) AMPA (A) receptors (GluARs), such that increases in activity are accompanied by global increases in GABA_A_Rs and decreases in GluARs (Nusser et al., 1998; Turrigiano et al., 1998; Kilman et al., 2002; Marty et al., 2004; Ibata et al., 2008). Indeed, we previously found that cholinergic basal forebrain neurons, which are active during waking and silent during SWS, responded to SD by increases in GABA_A_Rs (Modirrousta et al., 2007).

In the present study, we sought to determine if homeostatic changes occur in GABA_A_ and GluA1 receptors on excitatory cortical neurons of mice that are maintained awake during the day when they normally sleep the majority of the time. We employed unilateral whisker stimulation to enforce wakefulness and to be able to assess local, contralateral use-dependent changes in addition to bilateral sleep-wake state dependent changes in EEG activity and receptors in the somatosensory barrel cortex. We labeled cortical neurons with calcium/calmodulin-dependent protein kinase IIα (CaMK IIα) that has been shown to stain excitatory pyramidal and other neurons but not GABAergic inhibitory neurons in the cerebral cortex (Ouimet et al., 1984; Jones et al., 1994; Liu and Jones, 1996; Wang et al., 2013). Sections were dual-immunostained for the β2-3 subunits of the GABA_A_ receptor or the GluA1 subunit of the AMPA receptor or for the immediate early gene (IEG) proteins, c-Fos or Arc, which reflect enhanced activity with whisker stimulation (Filipkowski et al., 2000; Khodadad et al., 2015). Moreover, Arc has been shown to play a critical role in homeostatic synaptic scaling of AMPA receptors (Shepherd et al., 2006).

## Materials and Methods

All procedures were done in accordance with the Canadian Council on Animal Care and were approved by the McGill University Animal Care Committee. Twenty-eight adult C57BL/6 mice (20–25 g; Charles River Canada) were used in the study. The mice were housed individually and maintained on a 12/12 h light/dark cycle (lights on from 7:00 h to 19:00 h) at 22°C ambient temperature and with unlimited access to food and water in their cages.

### Video Behavioral and Telemetric EEG Recording

**Figure 1:**
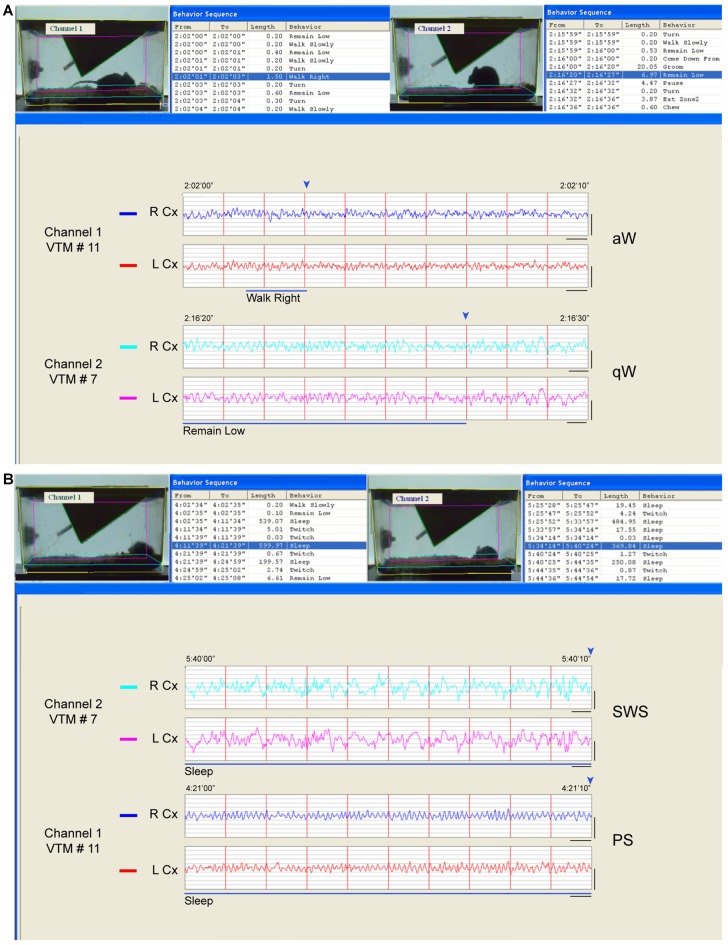
**Behavior and sleep-wake stage classification.** Behavior was classified automatically from video frames and sleep-wake stages visually from telemetric electroencephalographic (EEG). **(A)** Examples of wake behaviors and stages. In channel 1 (VTM #11), the video frame is fixed at the end (marked by blue arrowhead, above the EEG) of an episode, which was behaviorally classified as “walk right” (blue highlighted row, top left and blue bar, below) and which occurred during a 2 s epoch (and within a 10 s epoch) when the EEG activity was characterized by prominent high frequency beta and gamma (20.5–58 Hz) activity riding upon high theta activity (6.5–10 Hz) and was therefore classified as active or attentive Wake (aW). In channel 2 (VTM #7), the video is fixed at the end (marked by blue arrowhead, above) of an episode behaviorally classified as “remain low” (blue highlighted row, top right and blue bar, below) which occurred over multiple 2 s epochs (and within a 10 s epoch) when the EEG activity was characterized by less prominent high frequency activity riding upon low theta activity (4.5–6 Hz) and was therefore classified as quiet Wake (qW). **(B)** Examples of sleep behaviors and stages. In channel 2 (VTM #7), the video frame is fixed at the end (marked by blue arrowhead, above) of an episode which was classified behaviorally as “sleep” (blue highlighted row, top right and blue line, below) and which occurred during a 10 s epoch characterized by continuous prominent irregular slow wave activity in the delta range (0.5–4 Hz) and thus classified as slow wave sleep (SWS). In channel 1 (VTM #11), the video is fixed at the end (marked by blue arrowhead, above) of an episode which was also classified behaviorally as “sleep” (blue highlighted row, top left and blue line, below), which occurred during a 10 s epoch when the EEG was characterized by prominent rhythmic high theta activity (6.5–10 Hz) and thus classified as paradoxical sleep (PS). Screen display from HomeCageScan (Clever Systems) with which two mice were recorded simultaneously in adjacent cages. Records taken from baseline recordings. Calibration bars for EEG, vertical = 0.5 mV, horizontal = 0.5 s.

For scoring of sleep and waking, behavior was assessed by video recordings in 16 mice (VM) and behavior with EEG was assessed by video and telemetric recording in 12 mice (VTM; using Clever Systems, CleverSys behavioral recognition together with Data Sciences International, DSI, telemetry hard and software; Figure 1).

For EEG recording, mice were operated under anesthesia with isoflurane (4%–1.5% IsoFlo; Abbott Laboratories). Electrodes were placed bilaterally over the barrel cortex (Bregma−1.3 mm; lateral, ±3 mm; Paxinos and Franklin, 2001) and for reference, over the cerebellum. After being secured and covered with dental acrylic, their wires were run under the skin to the transmitter placed subcutaneously along the dorsal flank (F20-EET, DSI). The skin was sutured over the cemented electrodes and wires over the skull and neck. The VTM were allowed 1 week to recover from surgery.

During recording and experimentation, the mice were maintained in their home cages that were transferred to a separate room in the animal facility the mornings of the habituation, baseline and experiment. Behavior was recorded by video in VM and by video plus telemetry for EEG in VTM for up to 6 h in two mice at a time using HomeCageScan (HCS, 3.0; CleverSys). Each cage was placed in front of an artificial light panel for video recording and on a receiver (DSI) for EEG recording. All mice were given 2–3 days habituation to the recording situation prior to the experiment. At the end of this period, EEG was recorded in the VTM for 2 h between ~10:00 and 12:00 to obtain baseline EEG records.

### Sleep Deprivation and Recovery Experimental Procedures

**Figure 2:**
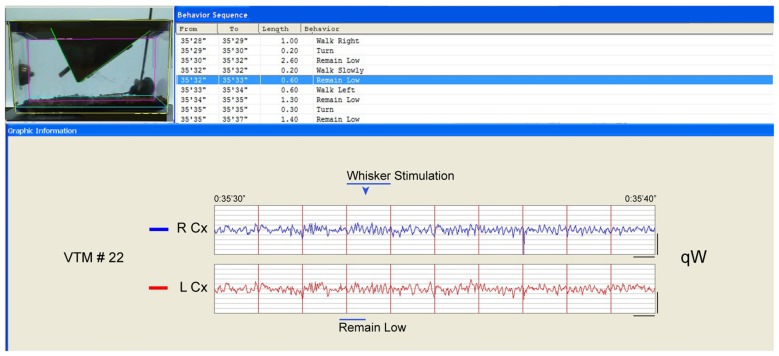
**Behavior and EEG changes elicited by whisker stimulation during sleep deprivation (SD).** As evident in the video frame (the time of which is marked by the blue arrowhead above the EEG), the whisker stimulation was performed using a soft brush inserted into the cage. In this case, the mouse had been in “remain low” or “walk slowly” then again “remain low” posture/behavior (blue highlighted row, top and blue bar., below) appearing to prepare to sleep. The whisker stimulation was then initiated and maintained for 1 s (bar) during which the animal moved (“walk left”). The EEG changed from irregular to rhythmic theta with gamma on both right (R) and left (L) parietal Cx during the stimulation of the left whiskers. After the stimulation, the mouse appeared to remain awake, while the EEG was characteristic first of qW followed by some aW such that this10 s period would be classified as qW. Calibration bars for EEG, vertical = 0.5 mV, horizontal = 0.5 s.

**Figure 3:**
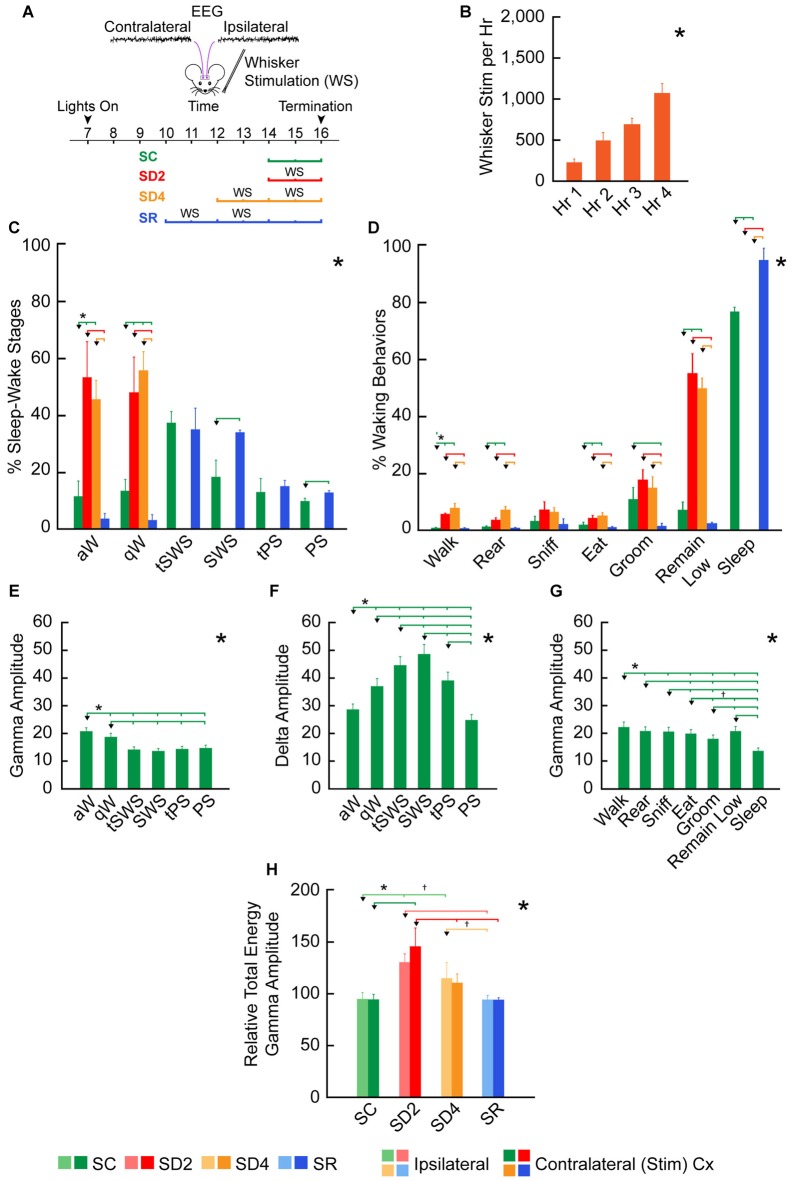
**Behavioral and EEG changes during SD and sleep recovery (SR).** **(A)** Experimental procedure using unilateral whisker stimulation to maintain mice awake during the day for 2 or 4 h of SD and preceding 2 h of SR. Mice were recorded by video alone (VM, *n* = 16) for behavior or video plus telemetry (VTM, *n* = 12) for behavior and EEG (over contra- and ipsilateral barrel cortices) for 2 h prior to termination (at ~16:00). They were divided into four groups: sleep control (SC), SD2, SD4 and SR (*n* = 4 VM and 3 VTM per group). **(B)** The number of whisker stimulations that were necessary to prevent mice from going to sleep increased significantly during 4 h SD (in SD4 and SR groups, *n* = 14). **(C)** The percentage of sleep-wake stages based upon video behavioral and telemetric EEG records in the four experimental groups (of VTM, *n* = 12) varied significantly across stages between groups. **(D)** The percentage of waking and sleep behaviors based upon video behavioral scoring together with EEG scoring in the four experimental groups varied significantly and differentially across behaviors between groups (of VTM, *n* = 12). **(E–G)** Average EEG gamma (30.5–58 Hz) and delta (0.5–4 Hz) integrated amplitude (mV per s) over the right barrel cortex varied significantly across and between sleep-wake stages **(E–F)** and behaviors **(G)** in baseline (of VTM, *n* = 12). **(H)** The relative total energy of gamma amplitude (calculated as percent of baseline value for each mouse) during the 2 h prior to termination in the four groups (of VTM, *n* = 12) varied significantly between groups on both sides of the barrel cortex. Large *indicates a significant (*p* < 0.05) main effect or interaction using one, two or three-way analyses of variances (ANOVAs; see text). Small *indicates a significant difference (*p* < 0.05) between the value on the left (marked by an arrowhead) and every other value to the right shown by a small vertical line for pairs connected by a horizontal bar, except where † indicates a trend (*p* < 0.10) of that difference between the pairs, using *post hoc* paired comparisons. Error bars indicate SEM.

The four experimental groups (4 VM and 3 VTM in each) were comprised by: (1) sleep control (SC) mice allowed to sleep undisturbed for 2 h; (2) sleep deprived mice maintained awake for 2 h (SD2); (3) sleep deprived mice maintained awake for 4 h (SD4); and (4) SR mice allowed to sleep for 2 h (SR) after being maintained awake for 4 h prior to euthanasia. SD mice were prevented from falling asleep by gently stimulating the left whiskers with a soft brush each time the animal showed signs of preparing to sleep (Figures 2, 3A). During habituation of all mice, the brush was placed in the home cage several times for familiarization though without touching the mouse. The experiments were conducted between ~10:00 (~ZT 3) and ~16:00 (~ZT 9) though shifted in time by ~30 min between mice in each pair (of SC and SD, SC and SR or SD and SR) to allow euthanasia of each mouse by the experimenter. At ~16:00 (~ZT 9), the mice were anesthetized with sodium pentobarbital (Euthanyl, 100 mg/kg i.p., Bimeda-MTC Pharmaceuticals) and perfused transcardially with 30 ml of cold saline followed by 200 ml of 3% paraformaldehyde solution. Brains were removed, post-fixed in 3% paraformaldehyde for 1 h at 4°C, then placed in 30% sucrose solution at 4°C for 2 days, frozen to −50°C and stored at −80°C.

### Behavioral and EEG Data Analysis

Behaviors were automatically classified from the video frames by HCS software (CleverSys; Figure 1). In HCS, 18 principal behaviors were scored: circle, walk (to right, left or slowly), turn, jump (jump or repetitive jump), stretch the body, rear up (come down, come down from partially reared, come down to partially reared, remain reared up, remain partially reared, rear up partially or rear up from partially reared up), sniff, eat (eat zone 1 or 2 or chew), dig, drink, forage, hang (hang cuddled, hang vertically, hang vertically from rear up, hang vertically from hang cuddled, remain hang cuddled, or remain hang vertically), groom, awake, remain low, twitch, pause and sleep. All behavioral classifications were verified manually. Settings for some behaviors were adjusted according to time. “Sleep” behavior was scored when the mouse maintained a sleep posture (with eyes closed) and did not move (for >900 frames or 30 s). Based upon behaviors which were most commonly present across groups of mice and represented more than 1% on average in all groups in baseline, we selected seven frequent behaviors: walk, rear up, sniff, eat, groom, remain low and sleep. In baseline recordings, the % frequent behaviors did not differ between the experimental groups.

EEG was analyzed in association with behaviors using the HCS software (CleverSys). Based on EEG and scored behaviors and using previously published criteria established in the rat (Maloney et al., 1997), we classified 2 s or 10 s epochs as one of six sleep-wake stages: active or attentive wake (aW), quiet wake (qW), transition to SWS (tSWS), SWS, transition to paradoxical sleep (tPS) and PS (Figure 1 and Supplementary Figure S1). During waking, when EEG and behaviors changed rapidly, 2 s epochs were scored as either aW or qW based upon EEG characteristics, whereas during sleep, when EEG and behaviors were more stationary, 10 s epochs were scored. For summary statistics, stages were also grouped into the three main states of wake (W, including aW and qW), non-rapid eye movement sleep (NREM, including tSWS, SWS and tPS) and REM sleep (corresponding to PS). The three distinct stages of aW, SWS and PS were scored when the EEG was characteristic for each stage during at least 65% (for aW) or 75% (for SWS and PS) of the epoch (Figure 1). The transitional stages of qW, tSWS and tPS were scored when the EEG was continuously intermediate in amplitude and frequency and/or in transition between distinct stages and characteristic of one stage for <65% or <75% of the epoch (Figure 1A and Supplementary Figure S1). aW was characterized by the predominance of high frequency (>20 Hz), low amplitude EEG activity along with rhythmic high theta activity (~6.5–10 Hz), the duration and frequency of which varied with specific waking behaviors, and qW by slower and slightly higher amplitude EEG activity which included irregular slow activity in a low theta range (4.5–6 Hz; Figure 1A). SWS was characterized by the predominance of irregular low frequency (~0.5–4 Hz), high amplitude EEG activity (Figure 1B), and the tSWS with less continuous or lower amplitude irregular slow activity (Supplementary Figure S1). PS was characterized by continuous rhythmic high theta activity (~6.5–10 Hz; Figure 1B), and tPS by less continuous or less rhythmic and lower frequency theta activity (Supplementary Figure S1). All epochs containing artifacts (representing on average 7% in baseline and 5% in experimental recordings) were excluded from the analysis. In baseline recordings, the EEG during different waking behaviors was characteristic of and thus scored as either aW or qW. The % aW scored was significantly higher than the % qW scored in all waking behaviors, except eating and grooming. In baseline recordings, the % of sleep-wake stages did not differ between the experimental groups.

Spectral analysis was performed on 2 s epochs using software specially designed for HCS (CleverSys) together with MATLAB R2014b (MathWorks). Output was computed and analyzed by integrated amplitude (mV) per frequency band: delta (0.5–4 Hz), theta 1 (4.5–6 Hz), theta 2 (6.5–10 Hz), sigma (10.5–15 Hz), beta 1 (15.5–20 Hz), beta 2 (20.5–30 Hz) and gamma (30.5–58 Hz). Average amplitude per s per stage or state was calculated per 2 h period. Total energy (average amplitude per s × number of s) was also calculated per frequency band per 2 h period (~7200 s). To normalize the data, we calculated the relative percentage of average amplitude or total energy during the experimental period (2 h pre-termination, ~14:00–16:00) with respect to that during the baseline recording (2 h ~10:00–12:00) in each animal. Comparisons were made using repeated measures (RM, within subjects) across three sleep-wake states, six sleep-wake stages, seven behaviors or two sides of the cortex and between measures (between groups) among the four experimental groups. These comparisons were applied using one-, two- or three-way analyses of variances (ANOVAs) with mixed RM followed by *post hoc* paired comparisons with Fisher’s LSD test or paired Student’s *t* test using Systat (v13). All values are expressed as mean ± SEM.

### Immunohistochemistry

Coronal sections were cut on a freezing microtome at 20 μm thickness and collected in 20 adjacent series at 400 μm intervals through the forebrain to include the anterior barrel cortex (Bregma 0.38 to −1.06; Paxinos and Franklin, 2001). The sections were processed for dual-immunostaining using the following primary antibodies: mouse anti-CaMKIIα (1:1000; Sigma-Aldrich Cat# C265 RRID:AB_258808) or rabbit anti-CaMKIIα (1:20; Thermo Fisher Scientific Cat# PA5-14315 RRID:AB_2070314); rabbit anti-c-Fos (1:10,000; Oncogene, Millipore Cat# PC38 RRID:AB_2106755); rabbit anti-Arc (1:1000; Synaptic Systems Cat# 156 003 RRID:AB_887694); mouse anti-β2-3 chain subunits GABA_A_R (1:100; Millipore Cat# MAB341 RRID:AB_2109419); rabbit anti-GluA1R (1:500; Millipore Cat# AB1504 RRID:AB_2113602); mouse anti-Rab5 (1:300; Abcam Cat# ab66746 RRID:AB_1141650) and rat anti-Rab11 (1:500; Abcam Cat# ab95375 RRID:AB_10688715). Most of the antibodies employed in this study were proven specific in their original development and applications (see RRIDs) and have been used extensively in published studies over many years. The mouse anti-CaMKIIα was characterized and employed in immunohistochemistry for identification of excitatory cortical neurons many years ago (Ouimet et al., 1984; Jones et al., 1994), and the rabbit anti-CaMKIIα yielded similar selective labeling in our application for immunostaining of pyramidal and other excitatory neurons in the barrel cortex. The rabbit anti-c-Fos and rabbit anti-Arc antibodies were both well characterized and have been in use for many years (Deurveilher et al., 2006; Modirrousta et al., 2007; Holloway and Mcintyre, 2011). The mouse antibody for β2-3 subunits of the GABA_A_R was produced and proven specific years ago and has since been used extensively in immunohistochemistry since it robustly stains the membrane of the most prevalent heteromeric GABA_A_ receptors on neurons in the brain (Fritschy and Mohler, 1995; Wan et al., 1997; Nusser et al., 1998; Modirrousta et al., 2007). The rabbit GluA1R antibody has been proven specific and utilized in immunohistochemistry in mouse cortex (Hagihara et al., 2011; Dixon-Salazar et al., 2014). In addition, we tested the specificity of this GluA1R antibody in immunofluorescent staining of barrel cortex in GluA1R knock-out mice (Mack et al., 2001) and found an absence of staining in those mice. Although some data for specificity are available for the mouse anti-Rab5 and rat anti-Rab11 (from Abcam, above), we tested their specificity for immunofluorescent staining of barrel cortex in our experimental mice and found that the staining could be blocked by pre-incubation of the antibodies with (100 fold concentration) immunogen peptide (for Rab5) or fusion protein (for Rab11, supplied by Abcam). Moreover, we found co-immunostaining of vesicular profiles, presumed endosomes with the mouse anti-Rab5 monoclonal and well characterized rabbit anti-Rab5 polyclonal (1:200; Abcam Cat# ab1821, RRID:AB_470264) antibodies. Similarly, we found co-immunostaining of presumed endosomes with the rat anti-Rab11 monoclonal and the well characterized rabbit anti-Rab11 polyclonal (1:200; Abcam Cat# ab3612 RRID:AB_10861613) antibodies.

Following rinsing in 0.1 M pH 7.4 trizma saline buffer and blocking in 6% normal donkey serum for 30 min at room temperature, the sections were incubated with combinations of two of the primary antibodies in buffer containing 1% normal donkey serum overnight at room temperature. Following subsequent rinsing, sections were incubated for 2 h at room temperature with combinations of appropriate secondary antibodies from donkey: Cy2-conjugated anti-rabbit (1:200; Jackson ImmunoResearch Labs, Cat# 711-225-152 RRID:AB_2340612), Cy2-conjugated anti-mouse (1:400; Jackson ImmunoResearch Labs, Cat# 715-225-150 RRID:AB_2340826), Cy3-conjugated anti-rabbit (1:1000; Jackson ImmunoResearch Labs, Cat# 711-165-152 RRID:AB_2307443), Cy3-conjugated anti-mouse (1:1000; Jackson ImmunoResearch Labs, Cat# 715-165-150 RRID:AB_2340813) or Cy5-conjugated anti-rat (1:800; Jackson ImmunoResearch Labs, Cat# 712-175-150 RRID:AB_2340671). After rinsing, sections were mounted, dehydrated using ascending concentrations of alcohols, cleared in xylene and cover slipped with Permount (Fisher).

### Immunohistochemical Image Analysis

Dual-immunostained sections through the barrel cortex were viewed by epifluorescent microscopy using a Leica DMLB microscope equipped with x-y-z motorized stage and digital camera (Orca-R^2^, C10600-10B, Hamamatsu photonics K.K.) with filters for Cy2, Cy3 and/or Cy5. Multichannel image stacks were acquired from CaMKIIα-c-Fos, CaMKIIα-Arc, CaMKIIα-GluA1R, CaMKIIα-GABA_A_R, Rab5-GluA1R or Rab11-GluA1R series and analyzed using StereoInvestigator software (MicroBrightField). Single- and double-labeled cell bodies or vesicular profiles (for Rab5 and Rab11) were mapped and counted in three sections (separated by 400 μm) from each dual-immunostained series using the Optical Fractionator Probe that allows unbiased, random sampling of elements within a defined volume. Contours were drawn around the barrel cortex (Paxinos and Franklin, 2001) in each section under a 2.5× objective and image stacks acquired with a 63× oil objective (1.4 numerical aperture). Optimal parameters were established for the sizes of the sampling grid (350 × 250 μm^2^) and counting frame (70 × 70 μm^2^) such as to best fit the contour of the barrel cortex and configuration of the cortical cells. Within each counting frame, paired image *z* stacks were acquired for two channels (of Cy2, Cy3 and/or Cy5) comprised by 0.5 μm thick optical sections through the mounted histological section of approximately 10 μm thickness. Within these images, all labeled cells or vesicles located 1 μm below the surface of the section were counted, thus through 9 μm of the section. Across the three sections, approximately 57 counting frames were acquired and analyzed per series. The estimates of the total number of CaMKIIα+ cells across the barrel cortex yielded a coefficient of error (CE, of Gundersen) of less than 0.1. The average estimated total number of CaMKIIα+ cells was 55,178 ± 8232 on the ipsilateral side and 42,000 ± 6690 on the contralateral side (across four series processed for dual-immunostaining with c-Fos, Arc, GluA1R and GABA_A_R). For the IEG proteins or receptors, positive immunostaining was determined according to established criteria for each protein (see “Results” Section) in the CaMKIIα+ cells and estimated total numbers of double-labeled cells were computed for each series (c-Fos+/CaMKIIα+, Arc+/CaMKIIα+, GABA_A_R+/CaMKIIα+ and GluA1R+/CaMKIIα+) and expressed as % of CaMKIIα+ cells per series through the barrel cortex. For the Rab proteins, the number of Rab5+/GluA1R+ and Rab11+/GluA1R+ double-labeled vesicles (presumed endosomes) were counted in each GluA1R+ cell on the contralateral side. Since one or two double-labeled vesicles were apparent in the control brains, the results were expressed as % GluA1R+ cells having two or more Rab5+/GluA1R+ or Rab11+/GluA1R+ vesicles.

For the GABA_A_R immunostaining, fluorescence intensity was measured over the plasma membrane of the GABA_A_R+/CaMKIIα+ cells on the contralateral side. As has been applied in previous *in vitro* studies (Ibata et al., 2008), the brightest immunofluorescent labeling over the membrane was selected for measurement in each of 10 GABA_A_R+/CaMKIIα+ cells per animal. The luminance measurements were performed on the cells in images that had been randomly acquired and analyzed using the Optical Fractionator probe (StereoInvestigator, above). For the acquisition, the same gain and exposure time were employed across all sections and brains using an 8-bit setting on the digital camera, thus providing converted gray scale images with arbitrary optical density units of 0–256. For measurement of receptor luminance, as previously employed (Toossi et al., 2016), a rectangular box sized at 1.5 × 0.3 μm^2^ was placed over the brightest immunostaining on the plasma membrane. Another box was placed over the nucleus of the same cell for measurement and subtraction of background staining in each cell. Luminance of the GABA_A_R membrane immunostaining was then compared across cells of the four groups.

Images were also acquired from sections dual-immunostained for CaMKIIα-GluA1R, CaMKIIα-GABA_A_R, Rab5-GluA1R or Rab11-GluA1R using a Zeiss LSM 710 laser scanning confocal microscope equipped with an Argon 488 nm, Helium-neon 543 nm and Helium-neon 633 nm for Cy2, Cy3 and Cy5 excitation respectively. Neurons were examined with a 63× oil objective (1.4 numerical aperture) and image stacks acquired with 360 nm thick optical sections. For publication, confocal images were adjusted in a consistent manner across groups for brightness and contrast and assembled in plates using Adobe Photoshop and Illustrator (Adobe Creative Suite, CS4).

The percentages of double-labeled cells in the barrel cortex were compared within the ipsilateral and contralateral sides between groups using two-way mixed RM ANOVAs followed by Fisher’s LSD paired comparisons or paired Student’s *t* tests (using Systat v13). Cell measures were correlated with sleep-wake and EEG measures using Pearson pairwise correlations (with Dunn-Sidak corrected probabilities for multiple correlations).

## Results

### Increasing Whisker Stimulation to Maintain Wakefulness during Sleep Deprivation

We employed unilateral whisker stimulation to prevent mice from going to sleep during the day (between ~10:00 and ~16:00), when they would normally be sleeping the majority of the time (Figure 3A). In this manner, we sought to account for homeostatic changes due to use, which should be specific to the contralateral barrel cortex, in addition to those due to enforced waking, which should be common to the ipsilateral and contralateral barrel cortices. We employed four experimental groups of animals, one (SC, *n* = 7), one sleep deprived for 2 h (SD2, *n* = 7), one sleep deprived for 4 h (SD4, *n* = 7) and one allowed SR (*n* = 7) for 2 h following 4 h SD, prior to termination for all groups at ~16:00. During SD, increasing numbers of whisker stimulations were necessary per hour to maintain the mice awake (for SD4 and SR groups using RM ANOVA, *F*_(3,59)_ = 53.20, *p* < 0.001; Figure 3B), reflecting increasing sleep pressure over 4 h SD.

### State Changes with Sleep Deprivation and Recovery

We assessed the effects of SD through behavioral scoring of video recordings in one set of mice (VM, *n* = 16) and through EEG scoring of telemetric recordings in addition to video recordings in another set of mice (VTM, *n* = 12; Figure 1). Through behavioral scoring in the two sets of mice, we established that the mice did not sleep in the sleep deprived (SD2 and SD4) groups and slept the majority of the time in the SC and SR groups in the 2 h period prior to termination at 16:00. Whereas there was no difference in baseline, the percentage of behavioral wake and behavioral sleep differed significantly in the experimental period between groups (ANOVA, *F*_(3,24)_ = 851.89, *p* < 0.001). The % sleep (of total recording time) was significantly greater in SR (mean ± SEM, 92.61% ± 2.21) than in SC (76.77% ± 2.56, *post hoc* paired comparisons, *p* < 0.001), indicating a homeostatic response to SD.

We also examined changes in sleep-wake states and stages along with behaviors in mice having both video behavior and telemetric EEG recording (VTM, *n* = 12, Figure 1). Whereas there was no difference between groups in baseline, the percentage of sleep-wake states, W, NREM sleep and REM sleep, varied significantly between groups in the 2 h experimental period prior to termination at 16:00 (two-way mixed RM ANOVA, interaction of state and group, *F*_(6,16)_ = 532.41, *p* < 0.001). The W state varied significantly between groups (one-way ANOVA, *F*_(3,8)_ = 818.45, *p* < 0.001). Both aW and qW stages varied (*F*_(3,8)_ = 17.17, *p* = 0.001 and *F*_(3,8)_ = 19.74, *p* = 0.001, respectively) and increased to a similar degree in SD2 and SD4 relative to SC (*post hoc*, *p* < 0.004; Figure 3C). The NREM sleep state (including tSWS, SWS and tPS) varied significantly (*F*_(3,8)_ = 389.02, *p* < 0.001), being absent in SD2 and SD4, and significantly increased in SR (82.29% ± 4.07) compared to SC (66.93% ± 1.71; *post hoc*, *p* = 0.001). The changes in NREM sleep state were due to changes in the SWS stage (*F*_(3,8)_ = 47.39, *p* < 0.001), which was significantly greater in SR (33.41% ± 0.81) than in SC (17.74% ± 4.61; *post hoc, p* < 0.001; Figure 3C). REM or PS also varied significantly across groups (*F*_(3,8)_ = 100.72, *p* < 0.001) and was significantly greater in SR (12.03% ± 0.87) than in SC (9.28% ± 0.89, *post hoc*, *p* = 0.014; Figure 3C). These changes in sleep-wake states or stages show the complete deprivation of sleep in SD2 and SD4 groups and the homeostatic rebound of sleep, specifically SWS and PS, in the SR group.

To examine in detail potential changes in behaviors, we selected and studied seven frequent video scored wake and sleep behaviors between groups (in VTM, *n* = 12). The percent of behaviors differed significantly across behaviors and differed significantly between groups (two-way mixed RM ANOVA, interaction of behaviors and group, *F*_(6,48)_ = 148.91, *p* < 0.001; Figure 3D). Although all waking behaviors tended to increase during deprivation, remain low increased to a greater degree than other behaviors (walk, rear, sniff, eat, groom), suggesting adoption of the most restful waking posture/behavior when prevented from sleeping.

### EEG Changes with Sleep Deprivation and Recovery

To assess changes in cortical activity across groups, we examined integrated amplitude in particular EEG frequency bands within the telemetric recorded mice (VTM, *n* = 12). In baseline recordings, gamma (30.5–58.0 Hz) amplitude varied significantly across stages (*F*_(5,55)_ = 59.0, *p* < 0.001), being maximal in aW and higher in aW and qW compared to all sleep stages, including PS (*post hoc*, *p* < 0.05; Figure 3E). Delta (0.5–4.0 Hz) amplitude also varied significantly across stages (*F*_(5,55)_ = 18.47, *p* = 0.001), being maximal in SWS and higher in SWS than in all other stages (*post hoc*, *p* < 0.003; Figure 3F). Gamma amplitude also varied significantly across frequent waking and sleep behaviors (*F*_(6,60)_ = 69.90, *p* < 0.001), being the highest during walking, lowest with grooming, intermediate with remain low during waking and the lowest overall during sleep (Figure 3G). These results indicate that gamma amplitude clearly reflects enhanced cortical activation during waking as distinguished from sleep and within waking during particular behaviors.

To assess changes in EEG activity across experimental groups, we examined amplitude in different frequency bands recorded in the experimental condition (~14:00–16:00) relative to that recorded in the baseline condition (~10:00–12:00) on a preceding day. Reflecting the changes in waking and sleeping, the relative average and total energy gamma amplitude during the 2 h prior to termination varied significantly on both sides of the barrel cortex (ipsilateral and contralateral to the whisker stimulation) between groups (two-way mixed RM ANOVA, *F*_(3,8)_ = 9.49, *p* = 0.005 with no significant difference or interaction with side; Figure 3H). The relative total energy gamma was most elevated compared to SC during the 2 h SD on both sides (one-way ANOVAs followed by *post hoc*, *p* < 0.05), though it was slightly higher on the contralateral side. With 4 h SD, gamma was lower than with 2 h SD and was not significantly different from SC on the contralateral side, suggesting a decrease in cortical activation and likely habituation after prolonged whisker stimulation and enforced waking. In SR, relative total energy gamma amplitude was not different from SC, suggesting a return to baseline/control levels. These changes in total energy gamma appear to reflect the changes in cortical activity, which occur with different sleep-wake stages and behaviors during deprivation and recovery (Figures 3C,D). In the wake state, differences in relative average gamma amplitude were evident between groups, yet not significant (two-way mixed RM ANOVA, *F*_(3,8)_ = 2.10, *p* = 0.178 with no significant difference or interaction with side), although the relative total energy gamma did differ significantly due to the increased time in waking. These results indicate that with the current paradigm of gentle whisker stimulation for preventing animals from falling asleep, the gamma activity during SD waking does not significantly exceed the normal or baseline/control levels. Similarly, the slight difference in gamma on the contralateral as compared to the ipsilateral cortex suggests that the whisker stimulation had a minimal effect above that of the stimulated arousal. When examining the EEG record (Figure 2), it appears that the whisker stimulation elicited a pattern of rhythmic theta with gamma activity, typical of aW, on both contralateral and ipsilateral sides. Following the stimulation, the EEG could return rapidly to a pattern of qW or a mix of qW and aW.

We also examined relative delta amplitude during the 2 h prior to termination to find that the average and total energy relative to baseline did not change significantly. This lack of significant change in relative delta activity during deprivation is likely due to the large increase during deprivation of qW, when delta is relatively high (Figure 3F). During the states of W, NREM and REM, we did not find significant differences in relative average delta amplitude between groups on the two sides of the cortex (two-way mixed RM ANOVA). In neither NREM nor specifically, SWS did we find a significant difference in relative average delta amplitude between SC and SR groups on the two sides of the cortex. These results suggest that the homeostatic adjustment to deprivation of NREM sleep occurred through an increase in the amount of NREM and specifically SWS, during which high amplitude delta activity is continuous and maximal in baseline/control (Figure 3F).

### CaMKIIα as Marker for Excitatory Cortical Neurons

**Figure 4:**
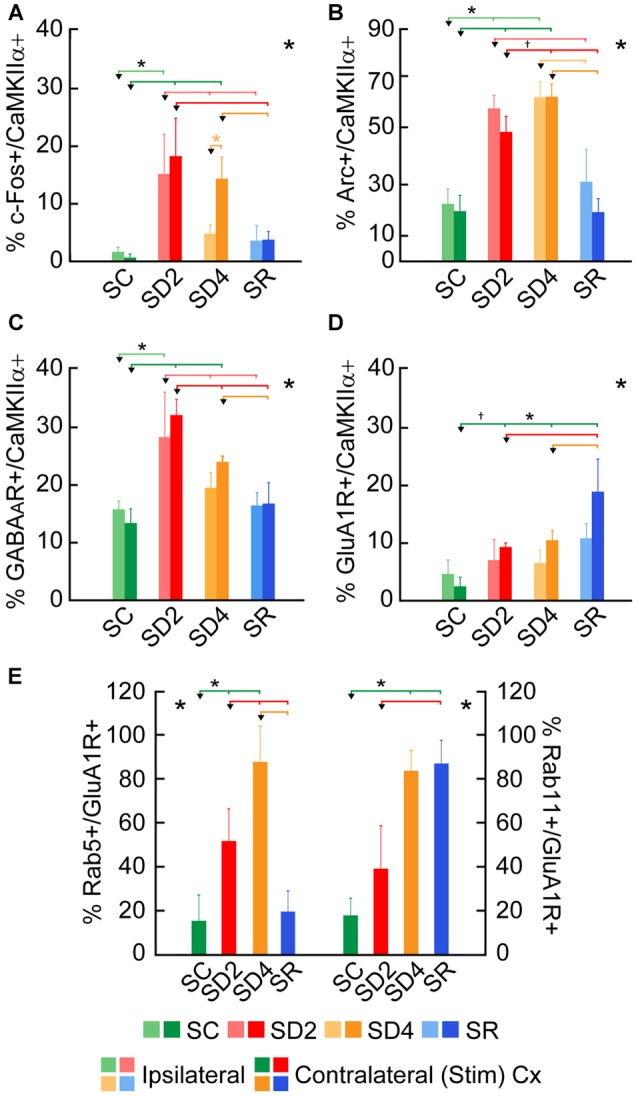
**Changes in expression of immediate early gene (IEG) proteins and receptors following SD and SR.** The percentage of cells that were considered double-labeled in dual-immunostained sections in the barrel cortex ipsilateral and/or contralateral to the whisker stimulation in the four groups of SC, SD2, SD4 and SR mice **(**Figures 5–10**)**. **(A)** Percent calcium/calmodulin-dependent protein kinase IIα (CaMKIIα)+ cells which were c-Fos+ varied significantly and differentially on the two sides of the cortex between groups. **(B)** Percent CaMKIIα+ cells which were Arc+ varied significantly on both sides of the cortex between groups. **(C)** Percentage of CaMKIIα+ cells which were GABA_A_ (β2-3 subunit) R+ varied significantly on both sides of the cortex between groups. **(D)** Percentage of CaMKIIα+ cells which were glutamate (GluA1) (subunit AMPA) R+ varied significantly between groups. **(E)** Percentage of GluA1 receptor (GluA1R)+ cells which were Rab5+ or Rab11+ on the contralateral side of the cortex varied significantly between groups. Large *indicates significant main effect of group using two-way ANOVAs with side **(A–D)** or one-way ANOVA for the contralateral side **(E)**, and small *indicates significant difference (*p* < 0.05) between the value on the left (marked by an arrowhead) and those indicated by a line and connected by bars to the right, except where † indicates a trend (*p* < 0.10), using *post hoc* paired comparisons (see text). *N* = 12 VTM in **(A–D)** and *n* = 12 VM and VTM in **(E)**. Error bars indicate SEM.

**Figure 5:**
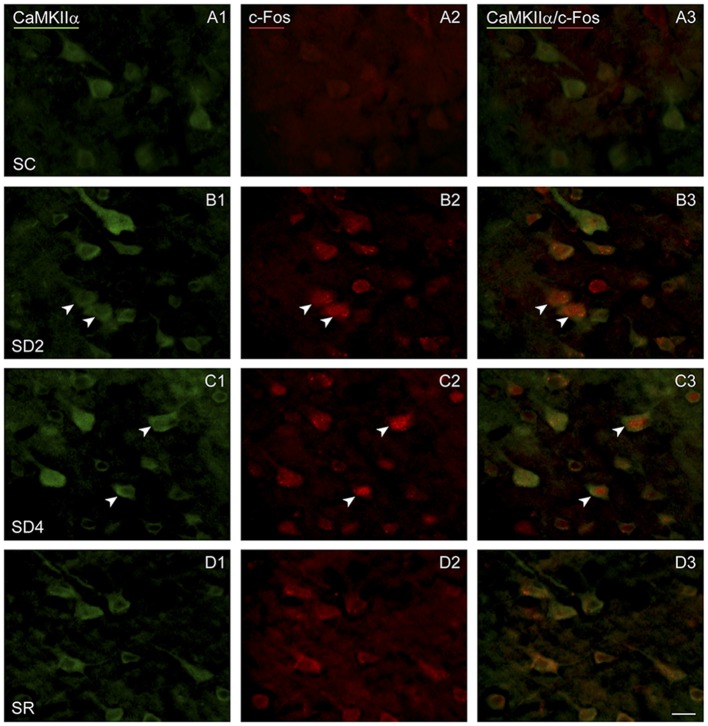
**c-Fos expression in CaMKIIα+ pyramidal cells following SD and SR.** Fluorescent microscopic images of sections dual-immunostained for c-Fos (red), as a marker of neuronal activity, and CaMKIIα (green), as a marker of pyramidal cells, in layer V of barrel cortex on the side contralateral to whisker stimulation. **(A)** In SC mouse, c-Fos immunostaining is not apparent in the CaMKIIα+ cells. **(B)** In SD2 mouse, c-Fos is apparent in the nucleus of CaMKIIα+ cells (arrowheads). **(C)** In SD4 mouse, c-Fos is still apparent in the nucleus of CaMKIIα+ cells (arrowheads). **(D)** In SR mouse, c-Fos is no longer apparent in the nucleus of CaMKIIα+ cells. Scale bar, 20 μm.

**Figure 6:**
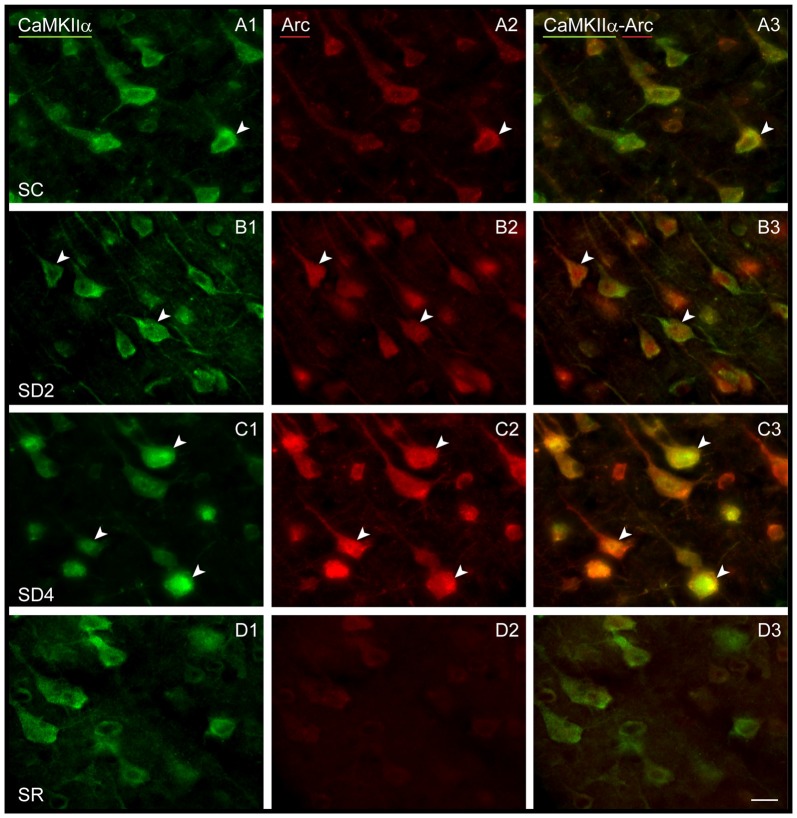
**Arc expression in CaMKIIα+ pyramidal cells following SD and SR.** Fluorescent microscopic images of dual-immunostained sections for Arc (red) and CaMKIIα (green) in layer V of the barrel cortex contralateral to whisker stimulation. **(A)** In SC mouse, Arc immunostaining is apparent in low intensity within the soma of some CaMKIIα+ neurons (arrowhead). **(B)** In SD2 mouse, Arc immunostaining is apparent in moderate to high intensity in the cytoplasm and nucleus of many CaMKIIα+ neurons (arrowheads). **(C)** In SD4 mouse, Arc is apparent in high intensity in many CaMKIIα+ neurons within the cytoplasm of soma and proximal dendrites and in the nucleus (arrowheads). **(D)** In SR mouse, Arc immunostaining is no longer apparent in CaMKIIα+-labeled neurons. Scale bar, 20 μm.

**Figure 7:**
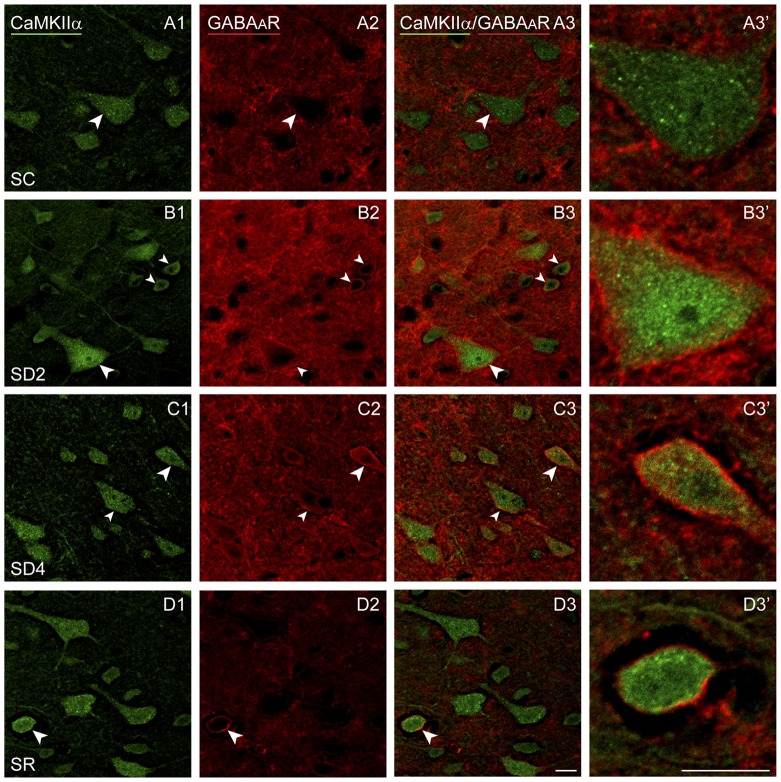
**GABA_A_Rs on CaMKIIα+ pyramidal cells following SD and SR.** Confocal images of sections dual-immunostained for (the β2-3 subunits of the) GABA_A_R (red) and CaMKIIα (green) in the layer V of the barrel cortex contralateral to the whisker stimulation. **(A)** In SC mouse, GABA_A_R immunostaining is apparent along portions of the plasma membrane in some CaMKIIα+ pyramidal cells (arrowhead). **(B)** In SD2 mouse, GABA_A_R immunostaining is apparent in high intensity along the entire plasma membrane of multiple CaMKIIα+ neurons (arrowheads). **(C)** In SD4 mouse, GABA_A_R immunostaining is still evident in high intensity along the entire membrane of CaMKIIα+ neurons (arrowheads). **(D)** In SR mice, GABA_A_R immunostaining is of relatively low intensity and apparent along portions of few CaMKIIα+ cells (arrowhead). Cells indicated with large arrowhead enlarged to right. Scale bars, 10 μm. Thickness, 1440 nm.

**Figure 8:**
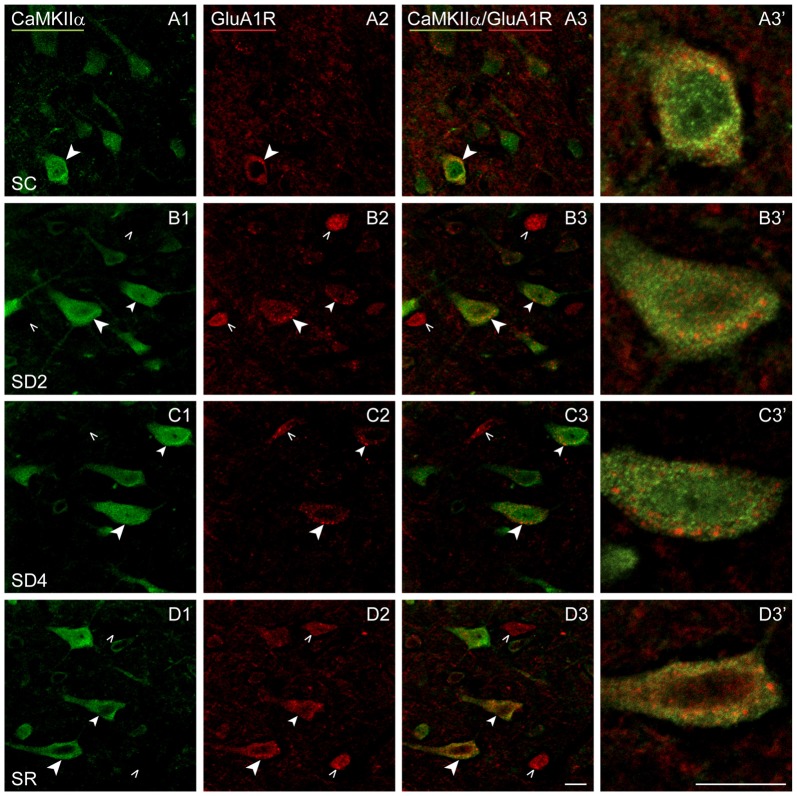
**GluA1Rs on CaMKIIα+ pyramidal cells following SD and SR.** Confocal images of dual-immunostaining for GluA1 (subunit of the AMPA) R (red) and CaMKIIα (green) in layer V of the barrel cortex contralateral to whisker stimulation. **(A)** In SC mouse, GluA1R immunostaining is apparent in relatively low intensity with a granular profile through the cytoplasm of few CaMKIIα+ cells (arrowhead). **(B)** In SD2 mouse, GluA1R immunostaining is apparent in medium intensity with prominent large granules in multiple CaMKIIα+ cells (arrowheads). **(C)** In SD4 mouse, GluA1R immunostaining appears less intense and concentrated within large granules within the cytoplasm near the plasma membrane in the CaMKIIα+ cells (arrowheads). **(D)** In SR mouse, GluA1R immunostaining is apparent in moderate to high intensity in small and large granules through the cytoplasm of soma and proximal dendrites of multiple CaMKIIα+ cells (arrowheads). Cells indicated with large arrowheads enlarged to right. Scale bars, 10 μm. Thickness, 3600 nm.

We used immunofluorescent staining for CaMKIIα, which marks pyramidal and other excitatory cortical neurons and not GABAergic interneurons (Jones et al., 1994; Wang et al., 2013), in order to visualize staining for IEG proteins and receptors in the excitatory neurons of the barrel cortex following SD and SR. CaMKIIα was visible in the cytoplasm and proximal dendrites of the pyramidal cells in layers, II/III, V and VI and in some nonpyramidal cells in layer IV of the barrel cortex. Using random sampling with an optical dissector tool, we mapped and quantified the CaMKIIα+ cells through all layers and on both sides of the barrel cortex in adjacent series of sections dual-immunostained for other proteins. We thus expressed the percent of CaMKIIα+ cells which were double-labeled for c-Fos or Arc (in 4 VM and 1 VTM sets, *n* = 20) or for GABA_A_R or GluA1R (in VTM, *n* = 12) across the four groups of mice (Figures 4–8). We established that there was no statistically significant variation in the number of CaMKIIα+ neurons between groups or with sides for any of the individual series (two-way mixed RM ANOVAs for group and side per series).

### Changes in IEG Proteins with Sleep Deprivation and Recovery

To examine enhanced neuronal activity as a function of whisker stimulation and enforced waking in the contralateral and ipsilateral barrel cortices, we employed immunofluorescent staining for c-Fos protein (Figure 5). In the control group (SC), no or little c-Fos staining was visible in CaMKIIα+ pyramidal cells in the cortex (Figure 5A). In SD2 and SD4 mice, c-Fos staining was evident in the nucleus and also the cytoplasm of CaMKIIα+ pyramidal cells in layer V (Figures 5B,C), as well as CaMKIIα+ cells in layers II/III, VI and IV. In SR mice, c-Fos was not visible in the nucleus though still evident to some degree in the cytoplasm of CaMKIIα+ cells (Figure 5D). CaMKIIα+ cells were considered c-Fos+ if the nucleus was positively stained. The number and proportion of CaMKIIα+ cells which were c-Fos+ was quantified on both sides of the barrel cortex in the four groups (from 4 VM and 1 VTM sets, *n* = 20). The proportion of c-Fos+/CaMKIIα+ cells differed significantly between groups (with two-way mixed RM ANOVA, *F*_(3,16)_ = 4.58, *p* = 0.017), between sides (*F*_(1,16)_ = 7.96, *p* = 0.012) and with a significant interaction between group and side (*F*_(3,16)_ = 5.14, *p* = 0.011; Figure 4A). These results suggest that c-Fos is differentially expressed across groups as a function of SD or SR and additionally between sides as a function of whisker stimulation or use. After 2 h SD, the percentage of c-Fos+/CaMKIIα+ cells increased markedly as compared to SC on both sides (one-way ANOVAs followed by *post hoc*, *p* < 0.03). After 4 h SD, the percentage decreased relative to SD2 on both sides (*post hoc*, *p* < 0.05), yet remained relatively high on the contralateral (stimulated) side, and decreased more markedly on the ipsilateral side (paired *t* test, *t* = −2.89, *p* = 0.044), suggesting continued c-Fos expression due to whisker stimulation on the contralateral but a homeostatic decrease in expression following prolonged enforced waking on the ipsilateral side. In the SR group, the proportion of c-Fos+/CaMKIIα+ neurons decreased from that of SD2 and SD4 on both sides to be not different from SC. Across animals (from VM and VTM sets, *n* = 20,), the % c-Fos+/CaMKIIα+ cells tended to be positively correlated with the number of whisker stimulations during the last 2 h in the contralateral barrel cortex (using Pearson’s pairwise correlation in two sets, *r* = 0.48, *p* = 0.061) and not in the ipsilateral cortex (*r* = 0.27, *p* = 0.425). It was more highly correlated with the % of behavioral waking in the contralateral cortex (*r* = 0.695, *p* = 0.001) and insignificantly in the ipsilateral cortex (*r* = 0.440, *p* = 0.102). The results suggest use and wake dependent expression of c-Fos.

We examined immunofluorescent staining for Arc protein, the product of another IEG whose expression is also stimulated by neural activity. In the control mice (SC), light Arc immunostaining was observed in the cytoplasm and primary dendrites of some CaMKIIα+ pyramidal cells in layer V (Figure 6A) and layers II/III and VI along with some cells in IV. In SD2 mice, moderate Arc immunostaining was present in the cytoplasm of the soma and primary dendrites and in the nucleus of CaMKIIα+ cells (Figure 6B). In SD4 mice, intense Arc immunostaining was evident in the cytoplasm of the soma and the dendritic branches and in the nucleus of CaMKIIα+ cells (Figure 6C). In SR mice, Arc immunostaining was barely visible in the cytoplasm and not visible in the nucleus (Figure 6D). The CaMKIIα+ cells were considered Arc+ with immunostaining of the cytoplasm in the soma. Arc+/CaMKIIα+ cells were plotted and quantified in the four groups (from 4 VM and 1 VTM sets, *n* = 20). The proportion of Arc+/CaMKIIα+ cells varied significantly between groups (with two-way mixed RM ANOVA, *F*_(3,16)_ = 15.78, *p* < 0.001) on both sides of the cortex (with no significant difference between sides or interaction with side; Figure 4B). In SD2, there was a significant increase in the % Arc+/CaMKIIα+ cells compared to SC on both sides (one-way ANOVAs per side with *post hoc*, *p* < 0.002). In SD4, the % Arc+/CaMKIIα+ cells appeared to remain high relative to SC on both sides (*post hoc*, p ≤ 0.005) or tended to increase further relative to SD2 on the contralateral side (*post hoc*, *p* = 0.074). In SR, it decreased to levels not different from SC. Across animals (from VM and VTM sets, *n* = 20), the % Arc+/CaMKIIα+ cells was significantly positively correlated with whisker stimulation on the contralateral side (Pearson’s, *r* = 0.75, *p* = 0.001) but also on the ipsilateral side (*r* = 0.72, *p* = 0.001) and more so with the % behavioral waking on both sides (*r* = 0.86 and 0.76, respectively, *p* < 0.001). These results suggest increases with Arc due essentially to enforced waking.

### Changes in GABA and Glutamate Receptors with Sleep Deprivation and Recovery

We examined immunofluorescent staining for the inhibitory GABA_A_R on CaMKIIα+ cells on both sides of the barrel cortex to determine if these receptors change as a function of whisker stimulation and waking across groups and sides. Immunostaining of the β2-3 subunits of the GABA_A_R was apparent on the plasma membrane of the CaMKIIα+ pyramidal cell bodies in layer V (Figure 7) and other CaMKIIα+ cells in layers II-III, VI and IV. In SC mice, GABA_A_R immunostaining was visible most commonly along portions of the membrane of some cells (Figure 7A). In SD2 and SD4 mice, the GABA_A_R immunostaining was more prominent and appeared to be present consistently along the full length of the plasma membrane of the soma and proximal dendrites of multiple cells (Figures 7B,C). In SR mice, the GABA_A_R immunostaining still appeared on the membrane though of fewer cells and most often along only portions of the membrane (Figure 7D). Across groups, CaMKIIα+ cells were considered GABA_A_R+ if the immunostaining was present along a portion or all of the membrane. The GABA_A_R+/CaMKIIα+ cells were mapped and quantified on both sides of the barrel cortex in the four groups of mice (from VTM set, *n* = 12). The proportion of CaMKIIα+ cells which appeared to be GABA_A_R+ varied significantly between groups (*F*_(3,8)_ = 12.66, *p* = 0.002) on both sides (with no significant interaction with side; Figure 4C), suggesting that GABA_A_Rs were altered mainly due to the SD. After 2 h SD, the proportion of GABA_A_R+/CaMKIIα+ cells was markedly increased relative to SC on both sides (*post hoc*, *p* < 0.006). After 4 h SD, the % GABA_A_R+/CaMKIIα+ cells decreased significantly compared to SD2 on both sides (Fisher’s, *p* < 0.03), yet remained significantly higher compared to SC on the contralateral side (Fisher’s, *p* = 0.006), suggesting a slight additional effect of whisker stimulation. In the SR group, the % GABA_A_R+/CaMKIIα+ cells came down to the SC level. Across these groups, the fluorescence intensity of the GABA_A_R immunostaining over the plasma membrane of the GABA_A_R+/CaMKIIα+ cells was measured on the contralateral side and found to vary significantly across groups (*F*_(3,116)_ = 7.33, *p* < 0.001) and to be significantly greater in SD2 than in SC (Fisher’s, *p* < 0.001). Across mice (*n* = 12 VTM), the % GABA_A_R+/CaMKIIα+ cells was significantly positively correlated on the contralateral side with whisker stimulation (Pearson’s, *r* = 0.76, *p* = 0.009) and not on the ipsilateral side (*r* = 0.50, *p* = 0.185). It was also positively correlated on the contralateral side with the % behavioral waking (*r* = 0.79, *p* = 0.017) and in a trend on the ipsilateral side (*r* = 0.59, *p* = 0.083). The % GABA_A_R+/CaMKIIα+ cells was most highly correlated on both contralateral and ipsilateral sides with the relative average total energy gamma amplitude (Pearson’s, *r* = 0.85, *p* = 0.002, *r* = 0.80, *p* = 0.01 respectively; Supplementary Figure S2). These results suggest that GABA_A_R changes predominantly as a function of waking and cortical activation, which is evoked by sensory stimulation.

We lastly examined the GluA1R. Immunofluorescent staining for the GluA1 subunits of the AMPA R was visible through the cytoplasm of the CaMKIIα+ pyramidal cells in layer V (Figure 8) as well as that of other cells in layers II/III, VI and IV. In SC mice, GluA1R immunostaining was seen in the cytoplasm extending from the nucleus to the periphery on or near the plasma membrane of the cell (Figure 8A). Within the cytoplasm, it was present in a light granular pattern but also concentrated in a few large granules or vesicles. In SD2 mice, GluA1R immunostaining was enhanced through the cytoplasm in more numerous large granules, which were located deep to the plasma membrane (Figure 8B). In SD4 mice, the GluA1R was most commonly seen in large granules, oftentimes close to the plasma membrane (Figure 8C). In SR mice, GluA1R immunostaining was seen throughout the cytoplasm of the soma and some primary dendrites and evident in large granules through the cytoplasm (Figure 8D). Across groups, the GluA1R immunostaining could not be adequately resolved over the plasma membrane. The CaMKIIα+ cells were thus considered positively labeled for GluA1R if immunostaining was present over the cytoplasm. The proportion of CaMKIIα+ cells which were GluA1R+ was quantified across groups (of VTM, *n* = 12). The percentage of GluA1R+/CaMKIIα+ cells varied significantly between groups (*F*_(3,8)_ = 6.89, *p* = 0.013) and by a trend between sides (*F*_(1,8)_ = 4.82, *p* = 0.059, with insignificant interaction between side and condition, *F*_(3,8)_ = 2.34, *p* = 0.150; Figure 4D). There was a progressive increase in the percent GluA1R+/CaMKIIα+ cells after 2 and 4 h SD as compared to that in the SC group, which reached significance on the contralateral side (*post hoc*, *p* = 0.076 and *p* = 0.044, respectively). In the SR group, the percent continued to increase on both sides to reach a maximum, particularly on the contralateral side (where it was higher than in all other groups, *post hoc*, *p* < 0.04). Across mice (*n* = 12 VTM), the % GluA1R+/CaMKIIα+ cells was not correlated with whisker stimulation or % behavioral waking. Given the ostensible changes in cellular distribution of GluA1R as well as in the proportion of double-labeled cells across groups, we questioned whether these changes reflected altered trafficking of the receptor.

To assess whether GluA1R might be trafficked differently within the cells between groups, we examined dual-immunostaining of the receptor with markers of different endosomes. Accordingly, series of sections from mice of each group were processed for immunofluorescent staining of GluA1R and Rab5, a marker of early endosomes, involved with internalization of receptors from the membrane (Bucci et al., 1992), or Rab11, a marker of recycling endosomes, involved with recycling receptors back to the plasma membrane (Ullrich et al., 1996). Given that there was no significant difference between sides or interaction of side with group in the % GluA1R+/CaMKIIα+ cells (above, Figure 4D), Rab5 and Rab11 immunostaining was analyzed on the contralateral side.

**Figure 9:**
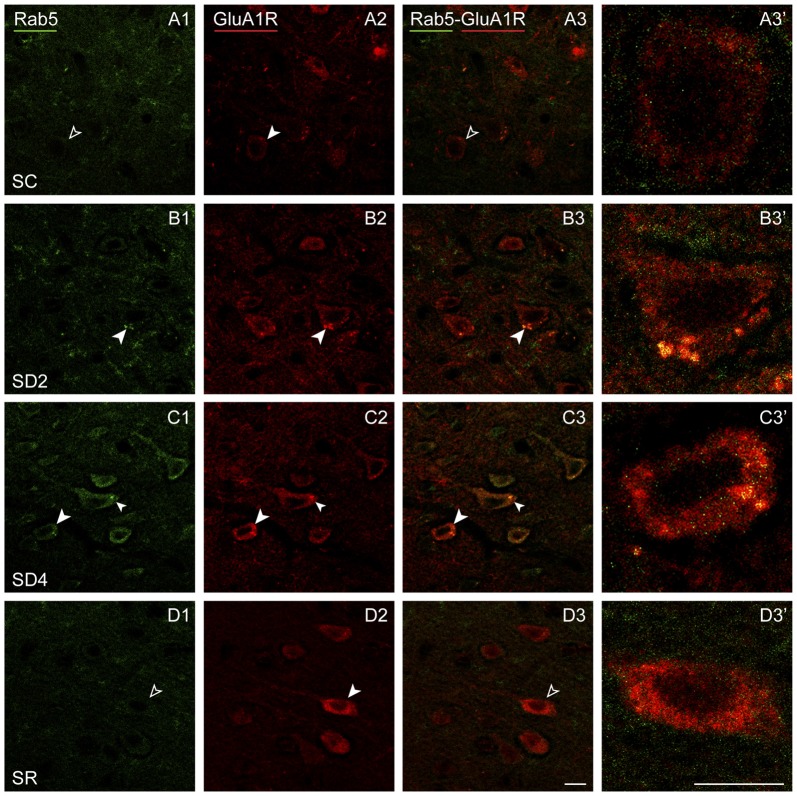
**GluA1Rs in early endosomes following SD and SR.** Confocal images of dual-immunostaining for GluA1 (subunit of the AMPA) R (red) and the marker of early endosomes, Rab5 (green), in layer V of the barrel cortex contralateral to the whisker stimulation. **(A)** In SC mouse, Rab5 immunostaining is just barely visible in a couple of endosomes which are lightly immunostained for GluA1R within a GluA1R+ cell (filled arrowhead), that was not judged to be Rab5+/GluA1R+ (unfilled arrowheads). **(B)** In SD2 mouse, intense Rab5 immunostaining is evident in multiple endosomes which are double-labeled for GluA1R (filled arrowheads) and are close to the plasma membrane in a GluA1R+ cell (filled arrowhead). **(C)** In SD4 mouse, Rab5 immunostaining is evident in multiple endosomes, which are double-labeled for GluA1R (filled arrowheads) in GluA1R+ cells (filled arrowheads). **(D)** In SR mouse, Rab5 immunostaining is not clearly visible in endosomes (unfilled arrowheads) within GluA1R+ cell (filled arrowhead). Cells indicated with large arrowhead enlarged to right. Scale bars, 10 μm. Thickness, 1440 nm.

Rab5 immunostaining was concentrated in large granules or vesicles presumed to be endosomes, which were dual-immunostained for GluA1R in certain groups (Figure 9). Rab5+ and GluA1R+ endosomes appeared to be negligible in SC mice, more prevalent in SD2 and SD4 mice, in which they appeared close to the plasma membrane, and negligible in the SR mice (Figures 9A–D). GluA1R+ cells were considered to be Rab5+ if they contained two or more double-labeled Rab5+ and GluA1R+ endosomes. The Rab5+/GluA1R+ cells were plotted and counted in the barrel cortex contralateral to the whisker stimulation in mice from the four groups (taken from 2 VM and 1 VTM sets, *n* = 12). The percentage of Rab5+/GluA1R+ cells varied significantly between groups (*F*_(3,8)_ = 11.04, *p* = 0.003; Figure 4E). Relative to SC, there was a progressive increase of % GluA1R+ cells containing double-labeled Rab5+ and GluA1R+ endosomes in SD2 and SD4 (*post hoc*, *p* < 0.04), reaching a maximum in SD4 and suggesting increasing internalization of the receptor during whisker stimulation and SD. In SR mice, Rab5+/GluA1R+ cells returned to levels not different from SC, which are minimal. Across mice (*n* = 12 VM and VTM), the % Rab5+/GluA1R+ cells on this, the contralateral side was highly correlated with whisker stimulations (*r* = 0.91, *p* < 0.001) and % behavioral waking (*r* = 0.77, *p* = 0.007).

**Figure 10:**
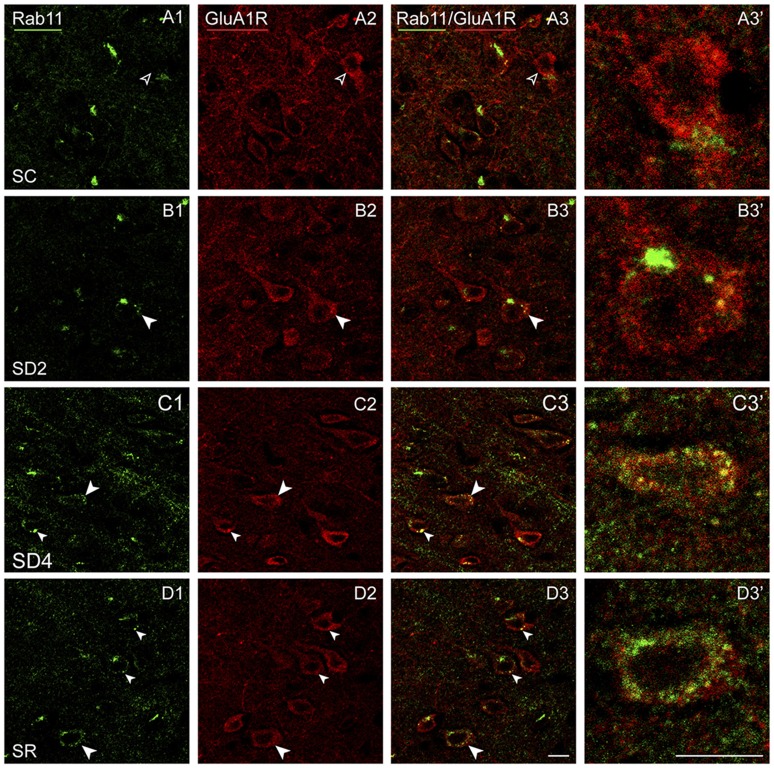
**GluA1Rs in recycling endosomes following SD and SR.** Confocal images of dual-immunostained sections for GluA1 (subunit of the AMPA) R (red) and the marker of late recycling endosomes, Rab11 (green), in layer V of the barrel cortex contralateral to the whisker stimulation. **(A)** In SC mouse, Rab11 immunostaining is not clearly visible in endosomes (open arrowheads) within GluA1R+ cell (filled arrowhead). **(B)** In SD2 mouse, Rab11 immunostaining is evident in one large clump, likely to be over the golgi apparatus, and in a few endosomes which are double-labeled for GluA1R (filled arrowheads) within GluA1R+ cell (filled arrowhead). **(C)** In SD4 mouse, Rab11 immunostaining is evident in many endosomes, which are double-labeled for GluA1R (filled arrowheads) and some close to the plasma membrane within GluA1R+ cell (filled arrowhead). **(D)** In SR mouse, Rab11 immunostaining is apparent in many endosomes, which are double-labeled for GluA1R (filled arrowheads) and some close to the plasma membrane within GluA1R+ cells (filled arrowheads). Cells indicated with large arrowhead enlarged to right. Scale bars, 10 μm. Thickness, 1440 nm.

Rab11 immunostaining was localized in large clumps, which appear to be over the golgi apparatus, and large granules or vesicles, which appear to correspond to endosomes (Figure 10). Rab11 colocalizes with the GluA1R in the endosomes, which become apparent with SD and SR. These Rab11+ endosomes which are GluA1R+ first appear in SD2 (Figure 10B) and then increase considerably in SD4 and SR through the cytoplasm extending out to the plasma membrane (Figures 10C,D). For mapping and quantification, GluA1R+ cells were considered Rab11+ if they contained two or more double-labeled Rab11+ and GluA1R+ granules (presumed endosomes) and were counted in the contralateral barrel cortex in the four groups of mice (taken from 2 VM and 1 VTM sets, *n* = 12). The percentage of GluA1R+ cells which were Rab11+ differed significantly between groups (*F*_(3,8)_ = 12.76, *p* = 0.002; Figure 4E). The % Rab11+/GluA1R+ cells increased progressively during deprivation, being significantly greater than in SC in SD4 mice (*post hoc*, *p* = 0.001). In SR mice, the % Rab11+/GluA1R+ cells remained at high levels similar to those in SD4 and significantly higher than those in SC and SD2 groups (*p* < 0.02). These results indicate that during SD4 and SR, GluA1R appears to be returned to the plasma membrane through an endosome recycling pathway in the vast majority of GluA1R+ cells. Across mice (*n* = 12 VM and VTM), the % Rab11+/GluA1R+ cells were not correlated with whisker stimulation or % behavioral waking. The results suggest that during 2 and 4 h SD, GluA1Rs are increasingly internalized from the plasma membrane into early endosomes, while they are subsequently redistributed into recycling endosomes through which they can be recycled back to the plasma membrane, particularly during SR when internalization appears to cease.

## Discussion

The present study shows that GABA_A_Rs are increased on the membrane of pyramidal and other excitatory cortical neurons in parallel with increases in high frequency gamma EEG activity during enforced waking. In contrast, GluA1Rs are internalized during waking and returned to the membrane during SR. The IEG proteins, c-Fos and Arc, show parallel changes suggesting a role for Arc in activity-dependent homeostatic scaling of the excitatory cortical neurons as a function of sleep-wake states and associated cortical activity.

### State and EEG Changes Induced by Sleep Deprivation with Sensory Stimulation

We aimed to study the effects of SD under the least stressful conditions possible. For this reason, we chose to perform the experiments within home cages and with unrestricted movement. We thus recorded and scored sleep and waking by video recording of behavior combined in half the animals with telemetric recording of EEG. We also aimed to deprive the animals of sleep by preventing them from falling asleep with gentle sensory stimulation and to be able to both define and quantify that stimulation in order to account for it and examine its potential induction of use-dependent changes. Such changes would be expected to be greater on the contralateral side than the ipsilateral side of the barrel cortex following stimulation of the left whiskers. We found first that the number of whisker stimulations necessary to maintain the animals awake increased significantly over 4 h SD indicating increasing sleep pressure. In examining the EEG, we found that the stimulation elicited increased rhythmic theta with gamma on the contralateral cortex during the stimulation, as shown in other studies (Hamada et al., 1999), but also on the ipsilateral side in association with the stimulated arousal. During the 2 h SD, we found that the relative average and total energy of gamma activity was significantly increased compared to control. However specifically during waking in the same period of SD, relative average gamma amplitude was not significantly increased compared to that in control. There was an equivalent increase in aW and qW EEG stages and increases in all waking behaviors but most of all in remain low behavior, during which the animal is still and accordingly appearing to rest. We thus believe that with the gentle whisker stimulation employed, the animals were minimally or not at all stressed during the deprivation. We also did not find significant differences in delta activity relative to baseline/control during recovery sleep on either the contralateral or ipsilateral side of the cortex, and thus did not find evidence of use- or state-dependent changes in delta as have been previously reported (Borbély et al., 1984; Tobler and Borbély, 1990; Vyazovskiy et al., 2000; Franken et al., 2001). We did however measure clear increases in the percent of the NREM state and specifically the SWS stage, when delta slow wave activity is maximal and gamma fast activity minimal, indicating a homeostatic response, which would serve to provide longer periods of rest for the cerebral cortex. The absence of changes in the amplitude of slow wave activity during SWS could be due to lack of stress, to relatively minimal and passive sensory stimulation or to the relatively short times of deprivation, since in other studies in rats, active, exploratory whisker stimulation on one side (after cutting whiskers on the other side) and 6 h SD were necessary for establishing significant increases in delta activity during SR (Tobler and Borbély, 1990; Vyazovskiy et al., 2000).

We also chose to use 2 and 4 h of SD since it has appeared that rodents can respond to the demand to stay awake during the day effectively in the short term but appear to have difficulty maintaining arousal and waking during longer periods. Notably, glutamate release in the cortex was found to increase in the short term of SD but to reach an asymptote around 3–4 h (Dash et al., 2009), and signs of local sleep begin to appear around 3–4 h of deprivation (Vyazovskiy et al., 2009, 2011). Here we found that relative total energy of gamma increased significantly after 2 h SD but less so after 4 h SD, likely reflecting the changes which occur with increasing sleep pressure with 3–4 h enforced waking. It is thus likely that the initial increase in cortical activation elicited by sensory stimulation and the induced waking is subsequently dampened by homeostatic changes.

### Use- and State-Dependent Changes in IEG Proteins

Corroborating previous reports (Filipkowski et al., 2000; Khodadad et al., 2015), we found that whisker stimulation caused expression of c-Fos and Arc in the contralateral barrel cortex during 2–4 h enforced waking. The proportion of c-Fos+/ CaMKIIα+ neurons differed between the stimulated and unstimulated sides, indicating that use-dependent activity added to wake-dependent activity in inducing this IEG. Whereas continuous whisker stimulation for 4 h maintained elevated c-Fos in the stimulated barrel cortex, the resulting continuous waking did not maintain increased c-Fos in the unstimulated cortex, suggesting a homeostatic reduction after an initial increase with continuous waking and cortical activation beyond 2 h. In contrast, Arc remained high on both ipsilateral and contralateral sides during 4 h enforced waking.

Both c-Fos and Arc have been known to be increased during waking and decreased during sleep in cortex and other regions (Cirelli et al., 2004; Maret et al., 2007). Translocated to the nucleus after 2–4 h enforced waking, as seen here, these IEGs may be involved in promoting synthesis of other factors involved in homeostasis and underlying the sleep-wake cycle (Krueger et al., 2008). Moreover, Arc is known to play a specific role in regulating homeostatic scaling of excitability through AMPA receptors (Shepherd et al., 2006; see below).

### Activity- and State-Dependent Changes in GABA_A_Rs

GABA_A_Rs appeared to increase on the plasma membrane of pyramidal and other excitatory cortical neurons following enforced waking and to return to baseline after SR. In our immunofluorescent material, the β2-3 subunit GABA_A_R immunostaining was apparent over the plasma membrane of only a small proportion (<15%) of the CaMKIIα+ cell bodies in control mice, presumably reflecting the minimal number in which the concentration of the receptor immunostaining reached the detection threshold for immunofluorescence. The GABA_A_R immunostaining became apparent here on a significantly (≥2X) larger proportion of the CaMKIIα+ cell bodies in sleep deprived mice, presumably reflecting an increased concentration of the receptor over the plasma membrane under these conditions. Supporting this assumption, the fluorescence intensity of the receptor immunostaining on the membrane was found to be significantly increased in the deprived mice.

The proportion of GABA_A_R+/CaMKIIα+ cells was most highly correlated with the relative total energy of gamma activity on both contralateral and ipsilateral sides of the barrel cortex. Like gamma activity and c-Fos, GABA_A_Rs reached a maximum after 2 h SD to decrease somewhat after 4 h SD, likely reflecting dynamic feedback in the homeostatic process. The changes in GABA_A_Rs seen here can be likened to those seen in neuronal cultures in which increased activity resulted in increased receptor clusters on the membrane (Marty et al., 2004). The GABA_A_R immunostaining here appeared to extend along the full plasma membrane of the CaMKIIα+ cell bodies and proximal dendrites after enforced waking. Such activity-induced increases in GABA_A_Rs have been shown *in vivo* in adult rats to correspond to mobilization of the receptor to the postsynaptic membrane and to be associated with an enhancement of inhibitory postsynaptic currents (Nusser et al., 1998). The increase seen here could also be associated with the extrasynaptic membrane since the β2-3 subunit chain of the GABA_A_R, as stained here, is present in both synaptic and extrasynaptic sites (Kasugai et al., 2010). Enhancement of GABA_A_Rs could thus result in increased phasic or possibly tonic GABA inhibition, which derives from extrasynaptic GABA_A_Rs (Mody et al., 1994; Brickley and Mody, 2012) and was recently shown to play a role in dampening cortical activation (Yu et al., 2015). Moreover, a chloride-mediated hyperpolarization through GABA_A_Rs has been shown to contribute to the initiation of silent (Off) states in the cortex (Lemieux et al., 2015). By such GABA inhibition, the increased cortical activation induced by enforced waking would be progressively dampened in what would thus correspond to homeostatic down-scaling that can occur in an autonomous manner in individual neurons with persistent increased activity (Turrigiano, 1999). The return to baseline during SR appears to reverse this process just as periods of inactivity in culture lead to decreases in GABA_A_R clusters on the membrane (Kilman et al., 2002; Marty et al., 2004). This process during SR would appear to correspond to homeostatic up-scaling (Turrigiano, 1999) that could return the neurons to their baseline level of excitability and activity.

### Activity- and State-Dependent Changes in GluA1Rs

GluA1 subunits of AMPA Rs changed in a different manner than the β2-3 subunits of GABA_A_Rs. First, the immunostaining was not concentrated over the plasma membrane of the pyramidal neurons but extended through the cytoplasm. With enforced waking, the immunostaining appeared to increase but particularly within large granules or vesicles in the cytoplasm, and with SR, it increased even more within these large granules, which on morphological grounds appeared to be endosomes. These changes were most apparent in the contralateral barrel cortex, though also present in the ipsilateral cortex, indicating use-dependent and state-dependent changes. We suspected that the qualitative and quantitative changes in the immunostaining likely reflected differential receptor trafficking within the cells during enforced waking and SR. We therefore examined dual-immunostaining of the GluA1Rs with markers for early or recycling endosomes. It was clear that during enforced waking with cortical activation, GluA1Rs were increasingly internalized by Rab5+ early endosomes, which remove AMPA receptors from the plasma membrane and synapses (Bucci et al., 1992; Brown et al., 2005). Rab5+/GluA1R+ neurons increased at 2 h to reach maximum at 4 h enforced waking and return to baseline levels after 2 h SR. These changes were correlated with both whisker stimulation and the percent waking. It thus appears that during sensory stimulation and enforced waking, GluA1Rs are internalized such that after 4 h, they could be reduced on the plasma membrane, as in synaptic down-scaling occurring in culture in response to prolonged increased activity (Turrigiano et al., 1998). On the other hand, GluA1Rs are also being progressively returned to the plasma membrane by Rab11+ recycling endosomes, which recycle AMPARs to the membrane (Ullrich et al., 1996; Park et al., 2004). This process appears to be initiated during 2 h SD and to increase with 4 h SD, presumably in working to maintain a functional level of receptors on the membrane during the prolonged waking and stimulation. However, whereas Rab5+/GluA1R+ cells decreased to baseline levels with SR, Rab11+/GluA1R+ cells continued to increase with SR, presumably permitting the full restoration of baseline levels of GluA1 receptors to the plasma membrane and synapses during sleep. Such reversible changes in homeostatic synaptic scaling involving AMPA receptors can be likened to those documented in culture (Turrigiano and Nelson, 2004) and *in vivo* in adult mice (Goel and Lee, 2007; Goel et al., 2011). The changes could involve extrasynaptic as well as synaptic AMPA receptors, which could also be associated with changes in excitability (Adesnik et al., 2005; Lu et al., 2009; Kopach et al., 2011; Granger et al., 2013).

The changes in GluA1R+/Rab5+ neurons paralleled changes in Arc+/CaMKIIα+ neurons, suggesting that Arc could play a role in the homeostatic regulation of GluA1Rs on the membrane. Indeed, Arc has been shown to play a critical role in homeostatic scaling by mediating endocytosis of the AMPA receptors in culture (Chowdhury et al., 2006; Shepherd et al., 2006). In addition, the translocation of Arc to the nucleus induced by prolonged activity *in vivo*, as seen here, has also been shown to decrease transcription of the GluA1R (Korb et al., 2013). The increasing expression of Arc during 4 h enforced waking could thus be responsible for down-scaling of GluA1R by endocytosis, whereas the return of Arc to baseline during SR would arrest this process. The unopposed exocytosis of the GluA1R could thus re-establish the excitability of the membrane of the excitatory cortical neurons during SR.

### The Role of Sleep in Neuronal Homeostasis

The present results suggest that enforced waking associated with increased total energy gamma activity during the day, when mice are normally asleep the majority of the time, induces homeostatic down-scaling through increases in GABA_A_Rs on the membrane of excitatory cortical neurons. SR associated with increases in SWS having maximal delta activity, permits the reduction of the membrane GABA_A_Rs and return to baseline levels. On the other hand, the sensory stimulation and enforced waking lead to internalization of GluA1Rs that can be mediated by increased Arc expression. Internalization is arrested during SR, when recycling of GluA1Rs to the membrane reaches maximal levels, suggesting restoration of baseline levels of AMPARs on the membrane. These reciprocal changes in GluA1Rs relative to GABA_A_Rs suggest down-scaling of excitability during prolonged enforced waking with high gamma EEG activity and up-scaling during sleep with slow waves to restore stable levels of excitatory and inhibitory receptors and resulting excitability and activity of excitatory cortical neurons.

These conclusions would be counter to those of the synaptic homeostasis hypothesis of sleep formulated by Tononi and Cirelli (2003). Indeed, with their colleagues, they have presented evidence for net synaptic potentiation during waking (Liu et al., 2010) and down-scaling during sleep through increased GluA1 receptor levels in cortex following natural or enforced waking as compared to sleep (Vyazovskiy et al., 2008a). Yet, the latter studies measured protein levels of the GluA1R in synaptoneurosomes of one cortical hemisphere by western blot and could not distinguish excitatory vs. inhibitory neurons, nor cell soma vs. dendrites or axons, nor differential trafficking of the receptor on or off the plasma membrane or synapse. On the other hand, another study which measured surface GluA1 and GluA2 subunits of the AMPA receptors in cortical neurons reported a decrease in surface expression of GluA1 subunits and an increase in that of GluA2 subunits after SD, indicating that GluA1-containing and GluA2-lacking, calcium-permeable AMPA receptors would be down-scaled during enforced waking with SD (Xie et al., 2016). In contrast using different techniques, studies examining the GluA1 and GluA2 subunits during dark vs. light periods of respective maximal wake vs. maximal sleep found somewhat different changes in synaptic AMPA receptors in cortex, likely in part due to the confounding of circadian and sleep processes (Lanté et al., 2011; Diering et al., 2017). But discrepancies could also be due to differential changes in synaptic versus extrasynaptic receptors on the membrane (Huganir and Nicoll, 2013). Here, our immunohistochemical results concerning GABAA and AMPA receptor subunits on the membrane suggest that for the major population of excitatory cortical neurons, homeostatic down-scaling occurs during prolonged stimulation and enforced waking and restorative up-scaling during recovery sleep during the light period. Such global homeostatic scaling does not preclude synapse-specific, Hebbian plasticity occurring during sleep or waking (Turrigiano, 1999).

Single unit recording studies have provided clear evidence for synaptic potentiation occurring during sleep (Aton et al., 2009, 2014; Chauvette et al., 2012). These studies have shown that synaptic potentiation and plasticity occur in association with slow wave activity and SWS and that SWS is essential for the plastic changes that occur in the shift of ocular dominance following monocular deprivation in kittens. Moreover, this plasticity during sleep was shown to depend upon enhanced glutamatergic transmission. On the other hand, recent studies in rats have found that recovery of unit discharge in visual cortex following monocular deprivation occurs during waking but not during SWS (Hengen et al., 2016). Yet, in that species with a fully crossed optic nerve, the recovery is from complete denervation, whereas in the kitten with a partially crossed optic nerve, the recovery represents a change in ocular dominance and thus plasticity within intact circuits (Aton et al., 2009). Indeed, the plasticity could depend upon the replay within intact circuits of the activity learned or occurring during preceding waking periods, as has been shown to occur during SWS (Wilson and Mcnaughton, 1994). In examining spontaneous activity within intact brains of rats, it has recently been found that pyramidal cell activity can increase or decrease during sleep depending upon its prior activity during waking, such that slow firing neurons would increase and fast firing neurons would decrease their rate of firing during sleep (Watson et al., 2016). GluA1 subunit receptor intensity on dendritic spines was also found by live cell imaging to vary up or down during the light-sleep maximum period depending upon their intensity during the preceding dark-wake maximum period (Diering et al., 2017). These results would fit with our results and a homeostatic model of neuronal activity as originally proposed by Turrigiano (1999), such that individual neurons would regulate their excitability and activity during sleep according to previous activity during waking and through global changes in excitability. Indeed, we recently found that SD induced an increase in GABA_A_Rs on orexin neurons, which fire during waking, whereas it induced a decrease in GABA_A_Rs on MCH neurons, which are silent during waking, followed by inverse restorative changes during SR (Hassani et al., 2009; Toossi et al., 2016). Increases in activity of individual neurons during waking can accordingly result in down-scaling during prolonged waking followed by up-scaling during recovery sleep to return to a stable level, as seen in our experiments involving population increases in activity during waking and decreases during recovery sleep here in cortical excitatory neurons. Such global homeostatic scaling could both preserve and allow for synapse-specific Hebbian synaptic plasticity during sleep as well as waking.

## Author Contributions

EC-P planned the research, conducted the research and analysis and participated in writing the manuscript. AP participated in conducting the behavioral and EEG experiments and analysis. LM performed the immunohistochemical processing of the brain tissue. BEJ planned the research, participated in the analysis of the data and writing the manuscript.

## Funding

The research was supported by a research grant (to BEJ) from the Canadian Institutes of Health Research (CIHR 13458).

## Conflict of Interest Statement

The authors declare that the research was conducted in the absence of any commercial or financial relationships that could be construed as a potential conflict of interest.
