# Supplementary material for: Homeostatic Changes in GABA and Glutamate Receptors on Excitatory Cortical Neurons during Sleep Deprivation and Recovery
Source: Front Syst Neurosci. 2017 Mar 31;11:17. doi: 10.3389/fnsys.2017.00017 (PMC5374161; doi:10.3389/fnsys.2017.00017)
Supplement: Supplementary file 1 [file Image_1.pdf]

Homeostatic changes in GABA and glutamate receptors on excitatory cortical neurons during sleep deprivation and recovery

Esther del Cid-Pellitero, Anton Plavski, Lynda Mainville and Barbara E. Jones
Barbara.jones@mcgill.ca

Supplementary Figure 1

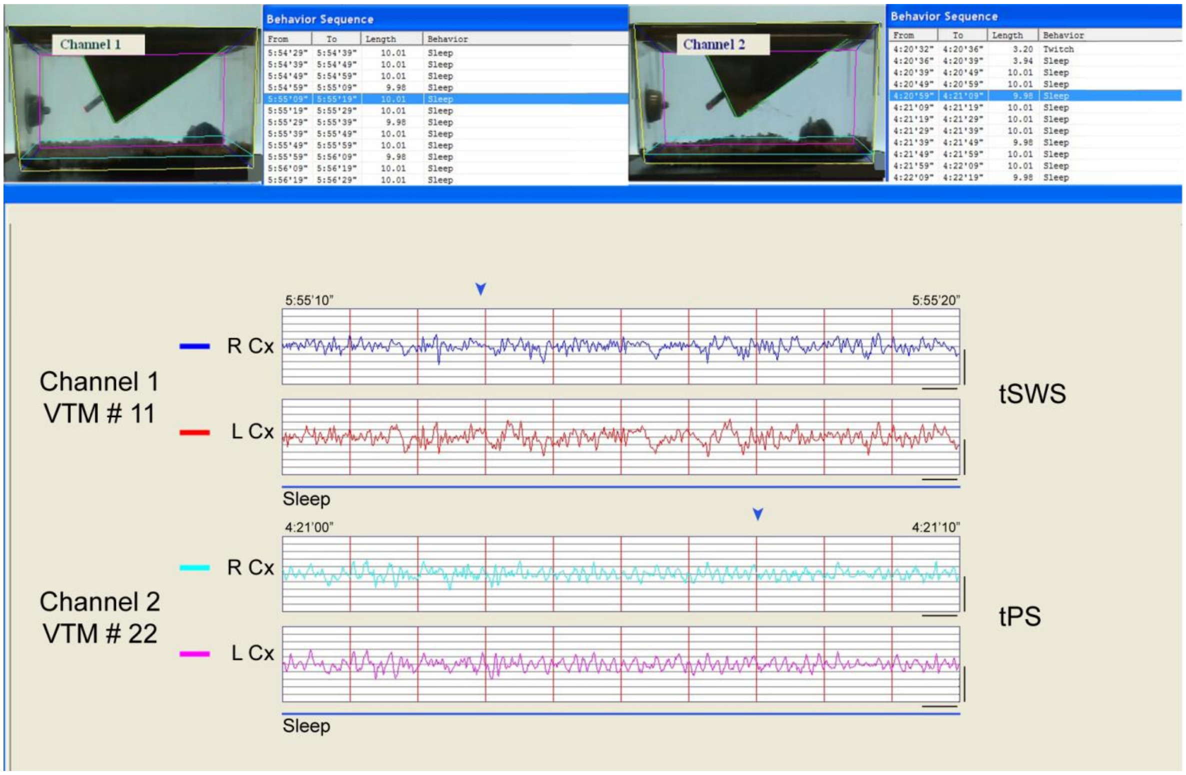

Transitional sleep stages. Examples of tSWS and tPS. In Channel 1 (VTM #11), the video frame is fixed at the end (marked by blue arrowhead, above) of a 10 s epoch, which was behaviorally classified as “sleep” (blue highlight, top left and blue line, below) and scored as tSWS according to the EEG activity which was characterized by irregular slow activity (0.5-4 Hz) occurring during less than 7.5 s and intermixed with spindle like activity. In Channel 2 (VTM #7), the video is fixed at the end (marked by blue arrowhead, above) of a 10 s epoch, which was also classified behaviorally as “sleep” (blue highlight, top right and blue line, below) and scored as tPS according to the EEG activity which was characterized by some rhythmic high theta activity (6.5-10 Hz) occurring during less than 7.5 s and intermixed with irregular low theta activity (4.5-6 Hz). Screen display from HomeCageScan (Clever Systems) with which two mice were recorded simultaneously in adjacent cages. Records taken from baseline recordings. Calibration bars for EEG, vertical = 0.5 mV, horizontal = 0.5 s.
