# Supplementary material for: Homeostatic Changes in GABA and Glutamate Receptors on Excitatory Cortical Neurons during Sleep Deprivation and Recovery
Source: Front Syst Neurosci. 2017 Mar 31;11:17. doi: 10.3389/fnsys.2017.00017 (PMC5374161; doi:10.3389/fnsys.2017.00017)
Supplement: Supplementary file 2 [file Image_2.pdf]

Homeostatic changes in GABA and glutamate receptors on excitatory cortical neurons during sleep deprivation and recovery

Esther del Cid-Pellitero, Anton Plavski, Lynda Mainville and Barbara E. Jones
barbara.jones@mcgill.ca

Supplementary Figure 2

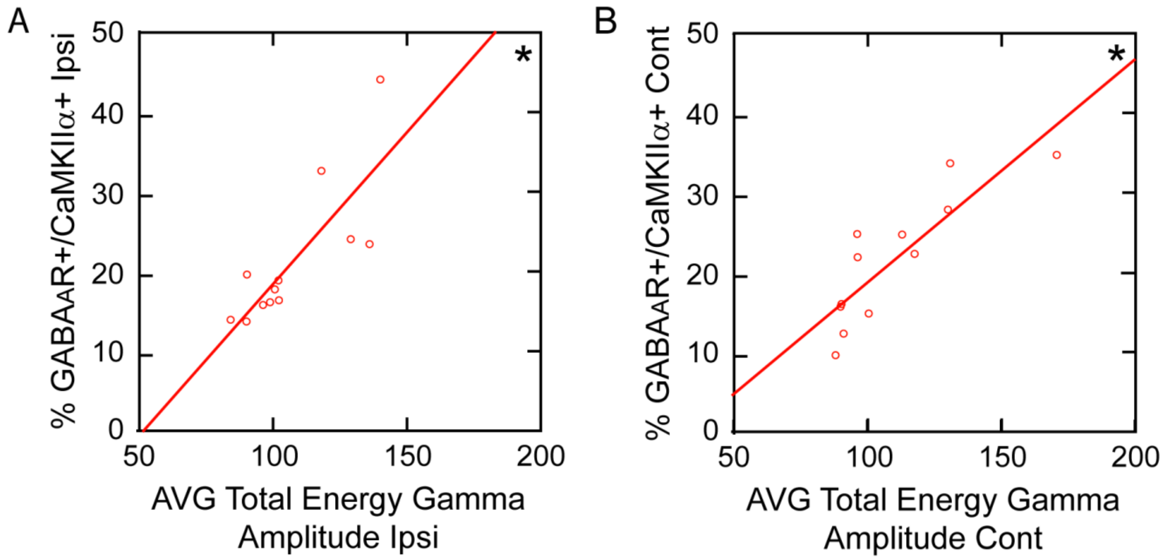

Supplementary Figure 2. Correlation between gamma EEG activity and $GABA_AR$+ cells across groups. The average relative gamma total energy on the ipsilateral (ipsi) and contralateral (cont) sides was plotted against the % CaMKIIα+ cells which were positively stained for the (β2-3) $GABA_AR$ on the respective sides of the cortex in individual animals. With Pearson pairwise analysis, the correlations were significant on both sides (*, $p < 0.05$, see Results).
